# How Officials’ Competitive Pressure Affects Sustainable Development Capacity From a Spatial Perspective: Empirical Evidence From China

**DOI:** 10.3389/fpsyg.2021.607232

**Published:** 2021-11-11

**Authors:** He Xu, Kun Wang, Guoping Li, Yufeng Zhang

**Affiliations:** ^1^Department of Investment, School of Management Science and Engineering, Central University of Finance and Economics, Beijing, China; ^2^Department of Accounting and Corporate Finance, Business School, Sichuan University, Chengdu, China; ^3^Department of High-Tech Business and Entrepreneurship, Faculty of Behavioural, Management & Social Sciences, University of Twente, Enschede, Netherlands; ^4^Department of Finance, Finance and Economics School, Jimei University, Xiamen, China

**Keywords:** local government, officials’ competitive pressure, sustainable development capacity, spatial analysis, entropy method

## Abstract

The view of political achievements suggests that officials will prefer to implement measures that benefit their own development in order to seek promotions. In the past, GDP weighed heavily in officials’ appraisals, leading them to develop the economy without regard to sustainability. Now that the central government has incorporated environmental indicators into the officials’ appraisal system, will this lead officials to implement sustainable development strategies to the fullest extent? Are there spillover effects and regional heterogeneity in this role? This paper discusses these questions with the help of entropy method and a spatial Durbin model using data of 30 provincial-level regions in China from 2006 to 2016. The conclusions show that, firstly, the officials’ competitive pressure is beneficial to enhance the sustainable development capacity of the province, but this effect is only effective in western China. Secondly, there is no spillover effect of officials’ competitive pressure on sustainable development capacity; thirdly, foreign direct investment, the proportion of state-owned enterprises and environmental regulations have their own unique effects on sustainable development capacity, and there are spillover effects. Based on these findings, this paper proposes corresponding policy recommendations in terms of officials’ appraisal system, talent training, foreign investment introduction, and state-owned enterprise reform.

## Introduction

The industrial revolution liberated productivity and greatly enriched the material life of human beings, but it was accompanied by a series of environmental pollution problems, which have become harmful to human life and survival. For example, industrial water pollution induces congenital malformations in infants (Dolk and Vrijheid, 2003), and plastic particles have been found in humans (Evangeliou et al., 2020). The growing ecological degradation and the awakening of environmental awareness determine that sustainable development will be a constant theme in the long term. The signing of the Rio Declaration provides a regulatory basis for this demand.

As China enters the 21st century, it is still a developing country whose economic development model is mainly driven by factor inputs. Therefore, the objective laws of economic development dictate that China’s economic development and ecological environment are in conflict with each other (Christmann and Taylor, 2001). For example, China’s economic development cannot be separated from the supply of carbon-based energy (Li et al., 2019; Tu, 2014), and the deepening urbanization inevitably generates environmental pollution (Liang and Yang, 2019). Of course, the Chinese government has responded positively to the world’s call for sustainable development by Equationting and enacting a series of timely policies to mitigate the conflict between economic development and ecological environment. For example, increasing environmental management costs and investment in forest greening (Sarwar et al., 2019), imposing environmental taxes (Ghazouani et al., 2020; Shahzad, 2020), encouraging green innovation (Guo et al., 2021), enhancing economic complexity (Doğan et al., 2021), encouraging diversified product exports (Shahzad et al., 2020, 2021), and adding environmental governance to the performance appraisal of local officials (Wang et al., 2019). Among them, an important policy that is of most interest to this study, and has not yet been fully examined, is the Chinese government’s inclusion of environmental indicators in the officials’ evaluation system.

Unlike capitalist countries, China implements a multi-party cooperation and political consultation system led by the Chinese Communist Party (CCP), which means that the CCP has far more power and influence than the parties in capitalist countries. This also means that Chinese local governments, which hold a large amount of political and economic resources, are able to intervene to a greater extent in the economic activities of firms (Jiang et al., 2012). For example, the quality of governance of officials in Russia, India and China after the 1990s caused significant differences in their economic performance (Bardhan, 2006). The corresponding reality is that during the first three decades of the Reform and Opening-up, the Chinese government focused its efforts on economic development, which in turn resulted in massive waste of resources and serious environmental pollution. During this period, local officials in China, in search of political achievements, have emerged the phenomenon of “judging heroes by GDP” (Zhou, 2007; You et al., 2018), i.e., the economic growth rate accounts for a large proportion of the officials’ assessment. This has led local governments to care only about economic development (GDP) and ignore problems such as inefficient resource use and environmental pollution (Yu et al., 2019). In conclusion, it is a clear fact that in order to enhance their own performance, local officials in China have a tendency to embark on governing activities (Li and Zhou, 2005), including environmental regulatory measures (Zhang et al., 2017). Therefore, when the weight of environmental protection increases in the officials’ appraisal system and the weight of economic development decreases, it is reasonable to assume that local officials will prefer to implement sustainable development policies.

An important question that is closely related to the above realities and policy context but has not yet been fully examined is: What is the relationship between officials’ competitive pressure (OCP) and sustainable development capacity (SDC) in China today? In other words, does OCP have a significant impact on SDC in China? If OCP has a significant effect on SDC, is this effect positive or negative? More importantly, are there spillover effects and regional heterogeneity in this impact? Exploring these questions, especially the spillover effects, has important policy implications for promoting sustainable development in China and winning the battle against pollution. These are the questions that this paper focuses on. Specifically, this paper examines these issues using a spatial econometric model with the policy change of including environmental indicators in the officials’ assessment system in China.

The remainder of this paper is organized as follows: Section “Literature Review and Hypotheses” compares and reviews the existing literature on the subject and presents the contribution and research hypotheses of this paper on this basis; Section “Methodology and Data” explains the contents related to the empirical evidence, including constructing regression models, selecting and measuring variables, and presenting the data; Section “Results” reports the main empirical results and regional heterogeneity results of official competitive pressure affecting sustainable development capacity and does robustness tests; Section “Discussion” discusses the conclusions obtained from this study; Section “Conclusion and Policy Recommendations” concludes the whole paper and makes policy recommendations.

## Literature Review and Hypotheses

The limited opportunities for upward mobility of local officials make political competition essentially a zero-sum game. The lack of incentives for cooperation among local officials and the incentives to “compare,” “compete” and “dismantle” each other make it impossible for officials to reach a win-win benefit compensation like businessmen do. This is the main reason for the existence of competitive pressure among local officials (Li and Zhou, 2005). Much of the early literature on officials’ competitive pressures (OCP) focused on behaviors and phenomena that explain local economic growth in China. It has been shown that economic performance-centered appraisal mechanisms exist at the provincial local government level in China (Zhou et al., 2005) and that the promotion rate of Chinese provincial officials is significantly and positively correlated with GDP growth rate (Li and Zhou, 2005). However, the main motivation for some local provincial officials to do their best to develop the economy is that the central government punishes provincial leaders who do not try to develop the economy rather than rewarding those who do (Duan, 2009). Moreover, it has also been argued that the promotion of local officials at the provincial level in China is primarily influenced by “connections” rather than economic performance (Opper and Brehm, 2007). While such findings seem to contradict existing political achievements view, at the root of the problem, we can find that the behavior of local officials has not changed. It has been shown that economic performance-centered appraisal mechanisms exist at the provincial local government level in China (Zhou et al., 2005) and that the promotion rate of Chinese provincial officials is significantly and positively correlated with GDP growth rate (Li and Zhou, 2005). However, the main motivation for some local provincial officials to do their best to develop the economy is that the central government punishes provincial leaders who do not try to develop the economy rather than rewarding those who do (Duan, 2009). Moreover, it has also been argued that the promotion of local officials at the provincial level in China is primarily influenced by “connections” rather than economic performance (Opper and Brehm, 2007). Such a conclusion seems to contradict the existing performance theory. However, at the root, we can find that the behavior of local officials has not changed. On the one hand, to avoid punishment and to obtain rewards only determine the purpose of local officials’ behavior (Yang et al., 2020) and do not change their behavior; on the other hand, even if they are promoted through “connections,” local officials still need to be fully committed to adequate economic performance and their preference for economic development remains unchanged. The preference for economic development remains the same. Therefore, the theory of performance is still valid.

Research on OCP in China has long been hot. In recent years, studies related to OCP have radiated to various fields, such as public facilities construction (Wu and Zhou, 2018), urban health and environmental governance (Liu et al., 2020), fiscal spending on science and technology innovation (Li and Li, 2020), local education spending (Jiang et al., 2017), and industrial pollution improvement (Zhang and Chen, 2018). And there is also a rich literature with firms as research subjects. Studies have found that competitive pressure from performance appraisal inhibits the development of corporate innovation (Cheng et al., 2020), and further, the higher the degree of financial decentralization, the stronger the inhibitory effect (Zheng and Lu, 2018). Not only that, OCP also significantly affects aspects such as firms’ indebtedness (Zhao, 2019) and resource allocation strategies (Xiong and Wang, 2017).

In addition, building sustainable development capacity requires various types of resources, of which capital and human resources are important components, but the limited amount of such resources may have a “siphon effect” on neighboring regions, which determines the externality of local governments’ behavioral measures. Some local officials may not care about sustainable development strategies at all, but are able to share the benefits of governance in neighboring provinces at zero cost. Thus, there is also a few literature that examines the spatial spillover effects of OCP (Yang et al., 2017; Li and Li, 2020). It is important to emphasize that while the sources of competitive pressure on officials mentioned in the literature differ, pressure from promotion incentives is significantly higher than that from age and tenure (Zeng and Zhu, 2021).

It is easy to find through the above literature that there is a wealth of research on OCP. Nevertheless, there is still room for expansion in the following important aspects. First, existing studies directly link OCP to environmental governance, but there are few studies on the relationship between OCP and sustainable development capacity. Second, the existing literature has examined fewer spillover effects arising from OCP, and the use of spatial econometric models is relatively arbitrary and lacks relevant testing steps. These are also the contributions of this paper on the basis of the existing literature. Based on the above arguments, this paper proposes the first two hypotheses to be tested.

Hypothesis 1 (H1): the officials’ competitive pressure from promotion incentives raises the level of sustainable development capacity in the province.

Hypothesis 2 (H2): there is a spatial spillover effect of the officials’ competitive pressure from promotion incentives.

Since China has distinctive regional characteristics in terms of economy, politics, and culture (Jiang and Yi, 2017), and this great regional variability is attributed to the unique locational characteristics of regions and the governance behavior of local governments (Zhou and Tao, 2011). Therefore, this paper further develops a third hypothesis.

Hypothesis 3 (H3): there is regional heterogeneity in the effect of officials’ competitive pressure from promotion incentives on sustainability.

## Methodology and Data

### Regression Model Setting

The purpose of this paper is to explore the impact and spillover effect of provincial officials’ competitive pressure on sustainable development capacity. Therefore, the initial empirical model constructed in this paper is shown in Equation 1.


(1) $$\ln S\,D\,C_{i\,t}=\theta +\alpha \,\ln O\,C\,P_{i\,t}+\beta \,\ln C\,o\,n\,t\,r\,o\,l_{i\,t}+\lambda_{t}\,\eta_{i}+\varepsilon_{i\,t}$$

In which, *i* presents province *i* and *t* presents period t. *SDC*_*it*_ is the sustainable development capability, *OCP*_*it*_ is the officials’ competitive pressure, and *Control*_*it*_ denotes control variables. *ε_*it*_* is random disturbance; *λ_*it*_* and *η_*it*_* reflects time-period fixed effect and spatial fixed effect, respectively.

Based on the previous discussion, the spillover effect among local officials mainly occurs in the surrounding areas of their jurisdictions, so this study uses a first-order adjacent spatial weight matrix to reflect this spillover effect and constructs a spatial Durbin model (SDM) form of Equation 1.


(2) $$\ln S\,D\,C_{i\,t}=\alpha_{1}\,\ln O\,C\,P_{i\,t}+\alpha_{2}\,W_{i\,j}\,\ln O\,C\,P_{i\,t}+\beta_{1}\,\ln C\,o\,n\,t\,r\,o\,l_{i\,t}+\beta_{2}\,W_{i\,j}\,\ln C\,o\,n\,t\,r\,o\,l_{i\,t}+\lambda_{t}+\eta_{i}+\varepsilon_{i\,t}$$

Equation 3 shows the form of the spatial weight matrix (*W*), while Equation 4 is the method of constructing the first-order adjacency spatial weight matrix (*W*_*ij*_), when i and j provinces are adjacent, *Wij* = 1; otherwise, *W*_*i*__*j*_ = 0.


(3) $$W=\left(\begin{array}{ccc} W_{11} & \cdots & W_{1\,j}\\ \vdots & \ddots & \vdots \\ W_{i\,1} & \cdots & W_{i\,j} \end{array}\right)$$

(4) $$W_{i\,j}=\left\{ \begin{array}{c} 1,P\,r\,o\,v\,i\,n\,c\,e\,i\,i\,s\,a\,d\,j\,a\,c\,e\,n\,t\,t\,o\,P\,r\,o\,v\,i\,n\,c\,e\,j\\ 0,O\,t\,h\,e\,r\,w\,i\,s\,e \end{array}\right.$$

### Variable Design and Measurement

#### Explained Variable

The explained variable in this paper is sustainable development capacity (SDC), which is defined in a number of ways, but the core ideas all emphasize the harmonious development of man and nature (Niu, 2012; Abson et al., 2017). Thus, SDC is a concept with rich connotations and researchers tend to measure it using comprehensive indicator methods. Such as, Fuzzy Analytic Hierarchy Process (Yu et al., 2017), Principal Component Analysis (Yang et al., 2013), Entropy Method (Tao et al., 2006), and Data Envelopment Analysis (Guo et al., 2016), etc. This paper expands on The Performance Evaluation Report of Chinese Green GDP (2018) to build an evaluation system of sustainability capacity in three dimensions: innovation, sustainability, and infrastructure (Table 1). The specific description of the evaluation system is as follows.

**TABLE 1:** Sustainable development capability evaluation system.

**Target indicator**	**Primary indicator**	**Secondary indicator**	**Unit**	**Average weight (2006–2016)**
Sustainable development capability	Innovation	R&D Capital Stock	Ten thousand yuan	11.50% (3rd)
		Full-time Equivalent of R&D Personnel	Man - year	9.80% (4th)
		Number of Patents Granted Per 10,000 People	Item / 10,000 people	14.76% (2nd)
		Financial Expenditure Intensity of Science and Technology	%	7.62% (6th)
	Sustainability	Technology Market Activity	%	22.59% (1st)
		Per Capita GDP	Yuan	7.24% (7th)
		Ratio of Tertiary Industry to GDP	%	7.16% (8th)
		Economic Energy Conversion Rate	Yuan / ton standard coal	4.67% (9th)
		Urban Sewage Treatment Rate	%	1.85% (12th)
	Infrastructure	Per capita road area	m_2_ / people	2.62% (10th)
		Green Coverage Rate in Built-up Area	%	2.16% (11th)
		Internet Penetration	%	8.02% (5th)

The innovation dimension aspect reflects the source of a region’s economic growth and is the key to achieving an innovation-driven development model. According to the general law of economic development, the economic growth mode of developing countries is dominated by the factor input-driven growth mode. However, on the one hand, the factor input-driven growth model suffers from low output efficiency and underutilization of resources, and its marginal scale efficiency is decreasing. On the other hand, non-renewable resources such as ore and oil are limited. Therefore, achieving an innovation-driven and intensive growth model has far-reaching implications for sustainable development. In this paper, four secondary indicators are used to reflect the level of innovation. (I) R&D capital stock (unit: Ten thousand RMB) reflects the amount of capital accumulation for R&D in science and technology. As capital is one of the decisive elements of output, R&D capital is also one of the decisive elements of innovative output. Sufficient scientific research funding supports the advanced equipment and materials of the R&D team and improves the efficiency of research and development. This study will use the Perpetual Inventory Method to process the data of R&D internal funds to measure R&D capital stock (Griliches, 1979; Tang et al., 2020); (II) Similar to R&D capital stock, R&D labor input is also one of the decisive factors for innovation output. This paper uses the full-time equivalent of R&D personnel (unit: person-year) to reflect the degree of R&D personnel full-time equivalent participating in scientific research; (III) innovation is a “step by step” process. A large amount of knowledge accumulation helps to improve the efficiency of R&D and eliminate obstacles to scientific research. Number of patent granted per 10,000 people (unit: item/10,000 people) reflects the number of innovative achievements with a certain value, and the degree of knowledge accumulation in a region; (IV) financial expenditure intensity of science and technology (unit: %) is the proportion of fiscal science and technology expenditures in total fiscal expenditures, which reflects the local government’s support for scientific research and reflects the government’s role in innovation and development.

The sustainability dimension reflects the current status of a region’s sustainable development. The Rio Declaration pointed out: For sustainable development, environmental protection should be a part of the development process and cannot be considered without it, which emphasizes the relationship between environmental protection and sustainability. Similarly, the Chinese government has long advocated the development principle—“Lucid waters and lush mountains are invaluable assets.” It can be said that environmental protection is a watershed that distinguishes sustainable development from traditional development. But, in reality, economic development will inevitably cause environmental pollution. Therefore, the key to sustainable development is to maximize economic output under the constraint of limited total amount of pollution. Methods such as developing the tertiary industry, researching and developing innovative products, and improving the utilization rate of resources can better deal with the above problems. This paper use five secondary indicators to reflect sustainability level. (I) Technology market activity (unit: %) is the ratio of transaction value in technical markets to GDP, which measures the degree of impact of the technology market on the entire market from the demand side. Products in the technology market tend to have higher profitability and resource utilization, which is conducive to improving regional sustainability; (II) per capita GDP (unit: Yuan) reflects the economic development level of a region. Because it is difficult to generate high profit margins with simple factor inputs, regions with higher per capita GDP have higher resource utilization. In addition, people with higher incomes have higher requirements on the quality of the environment (Iqbal et al., 2020), so that the government can improve the environment and increase the sustainable development capability in order to retain those people; (III) ratio of tertiary industry to GDP (unit: %) reflects share of the contribution of tertiary industry in economic market. Compared with the primary and secondary industries, the tertiary industry is environmentally friendly and resource-saving. Therefore, the higher the proportion of tertiary industry in a regional economy, the stronger its sustainability; (IV) economic energy conversion rate (unit: Yuan/ton standard coal) is the ratio of GDP to total consumption of energy, which measures the economic benefits that a unit of energy (energy produced by per ton of standard coal) can bring, and reflects the energy demand and energy efficiency of a region’s economic development from the side. Because economic development is difficult to get rid of energy constraints (Christmann and Taylor, 2001), the stronger the economic energy conversion rate, the stronger the sustainable development capability; (V) urban sewage treatment rate (unit: %) Reflect the Capability to treat domestic sewage in a region. Recycling is an important principle for sustainable development. Therefore, the higher the urban sewage treatment rate, the more beneficial it is to sustainable development capability.

The infrastructure dimension reflects the basic conditions of a region’s sustainable development. The infrastructure is a broad concept, including transportation, communications, medical treatment, health, education and other aspects. It is the foundation of social production and residents’ lives in the region, and it is also a necessary condition for the sustainable development of a region. As the economy enters the New Normal, China continues to move forward on the innovation-driven development road. Its requirements for infrastructure are not limited to traffic roads, but also have new requirements for communication and quality of life. Today, China has put forward the concept of “new infrastructure,” and infrastructure will play a greater role in sustainable economic development. This paper uses three secondary indicators to reflect the level of infrastructure. (I) Per capita road area (unit: m2 per person) not only reflects the amount of roads (length) in a region, but also reflects the quality of roads (width). The improvement of the quality and quantity of roads in a region can facilitate residents to go to work and travel, so as to attract enterprises to settle in, attract inflow of high-end talents, and promote sustainable development; (II) green coverage rate in built-up area (unit: %) reflects the greening level of the built area. Areas with high greening levels can make residents more attractive to high-end talents, thereby promoting sustainable development; (III) internet penetration reflects Internet users in a region. The Internet is the core facility in the digital age, which can facilitate the communication between people, improve the frequency and efficiency of communication, that is, save the cost of communication and cooperation and increase the efficiency of innovation, thereby promoting sustainable development.

**FIGURE 1:**
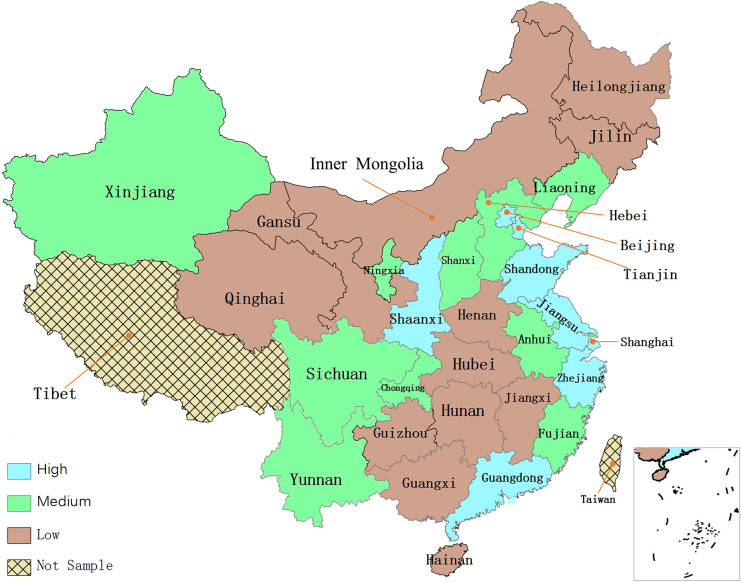
Geographical distribution of sustainable development capacity.

Entropy method assigns weights to secondary indicators based on the degree of variance among variables, and the greater the degree of variance, the higher its weight. It is very suitable for this paper as it can not only measure the complex SDC value, but also show the degree of contribution of each secondary variable. The operation process of entropy method can be referred to Tang et al. (2020). Figure 1 illustrates the spatial distribution of the mean values of SDC (2006–2016) for each province in China.

#### Core Explanatory Variable

Officials’ competitive pressure (OCP) is the core explanatory variable in this paper. According to the previous section, competitive pressure from promotion has the greatest impact on local officials (Zeng and Zhu, 2021). Therefore, this paper uses the turnover rate of provincial officials to measure OCP from promotion, following the method of Wang and Xu (2010). When the turnover frequency of officials at the provincial level increases, the uncertainty of their careers also increases, resulting in intense competition among officials, which is reflected in the improvement of officials’ performance. The steps for calculating OCP level are as follows.

Step 1. Set the intermediate variable (*M*_*it*_) to describe the external political competitive environment. See Equation 5 in details. The number of provincial regions included in the study in period *t* is *N*_*t*_; The total number of replacements of local officials (Only count the provincial party secretary and governor) in period *t* is *X*_*t*_. The number of replacements of local officials in province *i*, period *t* is *C*_*it*_.


(5) $$M_{i\,t}=X_{t}-C_{i\,t}$$

Step 2. Divide the intermediate variable (*M*_*it*_) by the number of provincial local officials that should be included in the statistical area that period, and obtain the official competitive pressure variable. See Equation 6.


(6) $$O\,C\,P_{i\,t}=\frac{M_{i\,t}}{2\,N_{t}}$$

#### Control Variables

The explanatory variable (SDC) in this paper is a comprehensive variable consisting of 12 variables from the three dimensions of innovation, sustainability and infrastructure. Therefore, this paper should avoid using too many control variables to prevent mutual causality between the independent and dependent variables, which may lead to endogeneity problems. For this reason, this paper only controls for three confounding factors: the influence from abroad, the influence from China’s specificity, and the influence from local officials’ sensitivity to environmental protection policies.

In this paper, we use foreign direct investment (FDI) to control for the influence from abroad. The advanced experience and technology brought by FDI helps to enhance SDC of the host country. Of course, there are some multinational companies that transfer low-tech, labor-intensive, and highly polluting industries to the host country, thus inhibiting the sustainable development of the host country.

This paper uses the nationalization rate (NR) to control for the effects from Chinese idiosyncrasies. Chinese state-owned enterprises (SOEs) are highly unique in that they operate with the primary goal of fulfilling social responsibility rather than profit maximization. In other words, at the call of the Chinese government, SOEs will actively work on energy conservation and environmental protection at any cost, thus improving sustainable development.

This paper uses environmental regulation (ER) to control for the influence from local officials’ sensitivity to environmental protection policies. China’s environmental governance strategy is planned by the central government and then implemented by local governments. This means that under the general framework set by the central government, local governments develop regulations based on the actual situation in their regions, i.e., they flexibly implement superior’s orders (Li and Zhou, 2005). Local officials who are sensitive to environmental protection policies will be more willing to implement the relevant measures, which will be reflected in the level of ER. Referring to Nie’s logic for measuring environmental regulations (Nie et al., 2021), the steps in this paper to measure ER are as follows.

Step 1. Calculate the emission intensity of pollutant v in province *i* in year *t* (*E*_*v, it*_). In which, *e*_*v, it*_ represents the total amount of pollutants *v*, and *Y*_*it*_ represents the actual industrial output value.


(7) $$E_{v,i\,t}=\frac{e_{v,i\,t}}{Y_{i\,t}}$$

Step 2. Calculate the national environmental pollution emission intensity *NE*_*v, it*_:


(8) $$N\,E_{v,i\,t}=\sum_{i=1}^{30}\frac{e_{v,i\,t}}{Y_{i\,t}}$$

Step 3. Calculate a comprehensive environmental pollution index of local government (*EP*_*it*_), which represents the relative value of the emission intensity of environmental pollution of the province *i* in the period *t*.


(9) $$E\,P=\frac{1}{3}\,\frac{E_{v,i\,t}}{N\,E_{v,i\,t}}$$

Step 4. The larger EP is, the more the environmental pollution is, which indicates that environmental regulations have deteriorated. In order to be consistent with the expected coefficient of the theoretical hypothesis, we reversed the Equation 9 and get environmental regulation (*ER*_*it*_) variable. This means the higher ER is, the higher the government’s environmental regulation is.


(10) E⁢R=1/E⁢P

### Data

Use panel data from 30 provincial regions in China from 2006 to 2016. To ensure the integrity of the original data, the Tibet Autonomous Region, Hong Kong, Macau and Taiwan were excluded. All raw data is collated from China Statistical Yearbook, China Statistical Yearbook on Science and Technology, Provincial Statistical Yearbook. Besides, the raw data of officials’ changes are collated from the Internet^1^. For the few missing data, use the moving average method to fill in, which does not affect the overall evaluation results. Table 2 shows the descriptive statistics of each variable.

**TABLE 2:** Descriptive statistics.

**Variable**	**Unit**	**Obs**	**Means**	**Std.**	**Max**	**Min**	**Label**
Sustainable development capacity	%	330	24.60	15.69	86.78	3.86	SDC
Official competitive pressure	%	330	26.66	14.46	55.00	10.00	OCP
Foreign direct investment	10,000 dollars	330	655,450.7	716,940.70	3,575,956	1,495	FDI
Nationalization rate	%	330	62.51	18.36	98.13	16.63	NR
Environmental regulation	%	330	50.35	49.70	285.32	3.86	ER

## Results

### Empirical Results

Elhorst (2014) pointed out that the regression coefficients of the spatial Durbin model (SDM) do not have explanatory power and should be decomposed into direct and indirect effect coefficients for interpretation. Table 3 reports the regression results of Equation 2. It can be found that the direct effect coefficient of officials’ competitive pressure (OCP) is 0.6608 and passes the significance test with a significance level of 10% (H1 holds), indicating that the higher the OCP is, the more it is conducive to the improvement of the province’s sustainable development capacity (SDC). Specifically, every 1% increase in OCP level will increase the SDC level of this province by 0.6608%. However, the coefficient of the indirect effect of OCP fails the significance test with a significance level of 10%, indicating that local OCP has no significant effect on the SDC of neighboring provinces, i.e., there is no significant spillover effect (H2 does not hold). As for the control variables, the regression results for foreign direct investment, nationalization rate, and environmental regulation are good, and the coefficients of direct and indirect effects are mostly significant and their coefficient signs are more consistent with the previous expectations, indicating that they play the role of control variables.

**TABLE 3:** Direct and indirect effects of China.

**Direct effect**				**Indirect effect**			
	**Coe**	**Robust Std.**	***P*-value**		**Coe**	**Robust Std.**	***P*-value**
OCP	0.6608	0.3776	0.080	OCP	0.0598	0.1087	0.582
FDI	0.1853	0.0483	0.000	FDI	–0.1031	0.0602	0.087
NR	0.3172	0.0531	0.000	NR	0.3723	0.1366	0.006
ER	0.1075	0.1198	0.369	ER	0.4553	0.2109	0.031
*R* ^2^	0.5845						

### Robustness Test

#### Model Fixed Effects Test

There are three forms of panel model fixed effects: time-period fixed effect, spatial fixed effect and spatio-temporal (S&T) fixed effect. Among them, the S&T fixed effect has the widest applicability. Therefore, it is used by default in Equation 2. To make the empirical regression results more convincing, these three fixed effects are determined by F-test in this paper. In addition, SDM has wider applicability than spatial error model (SEM) and spatial lag model (SLM), so it is used by default in Equation 2. In this paper, the Wald test will be used to determine whether the SDM can be simplified to SEM or SLM. The results of the above tests are reported by Table 4. Table 4 shows that the model of Equation 2 has S&T fixed effect and SDM cannot be simplified, which also proves that the reversion model of this study is optimal.

**TABLE 4:** Regression model test.

**Null hypothesis**	***F*-value**	**Wald-value**
Null hypothesis: no spatial fixed effect	3.95***	–
Null hypothesis: no time-period fixed effect	29.60***	–
Null hypothesis: no S&T fixed effect	22.54***	–
Null hypothesis: SDM can be simplified to SEM	–	28.57***
Null hypothesis: SDM can be simplified to SLM	–	79.24***

***Indicates statistical significance at the 1% levels.

#### Spatial Auto-Correlation Test

Tobler’s First Law of Geography suggests that “everything is related to everything else, but near things are more related than distant things” (Tobler, 1970). The presence of spatial auto-correlation of the explanatory variables is a necessary precondition for the use of spatial econometric models.

Moran’s I test is the most commonly used global spatial auto-correlation analysis method, which can investigate the spatial aggregation of the entire spatial sequence. The closer Moran’s I statistic is to 0, the weaker the spatial auto-correlation is, and the closer to 1 or −1, the stronger the spatial auto-correlation is. Among them, if the sign is positive, it means positive auto-correlation, and the negative sign means negative auto-correlation. The Moran’s I statistics of SDC from 2006 to 2016 were measured by StataSE 15, as shown in Table 5. It can be found that only in 2007 and 2011, the *p*-value is greater than 0.1, which means that it has not passed the significance test, but does not affect the overall statistical situation. So, we judge that there is a significant positive spatial auto-correlation in sustainable development capability.

**TABLE 5:** Moran’s I statistics of sustainable development capacity.

**Year**	**Moran’s I**	***P*-value**
2006	0.205	0.029
2007	0.110	0.195
2008	0.264	0.007
2009	0.251	0.009
2010	0.232	0.017
2011	0.109	0.204
2012	0.335	0.001
2013	0.297	0.003
2014	0.227	0.017
2015	0.274	0.006
2016	0.259	0.012

**FIGURE 2:**
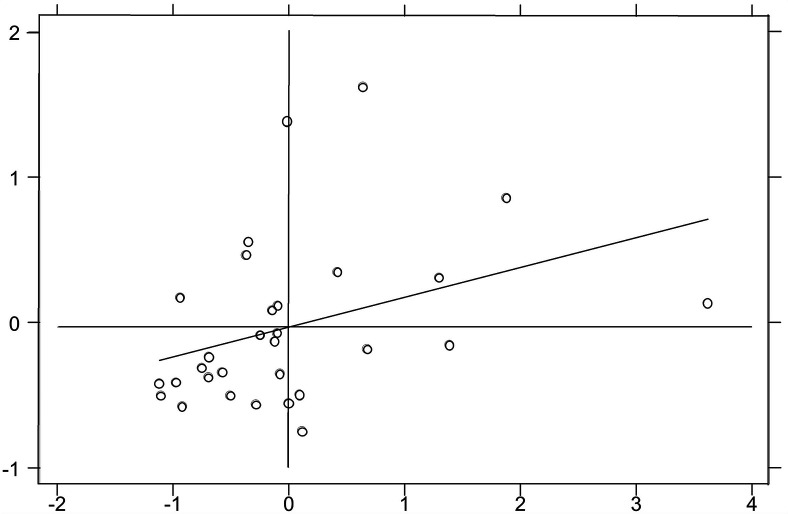
Moran scatter plot in 2006.

**FIGURE 3:**
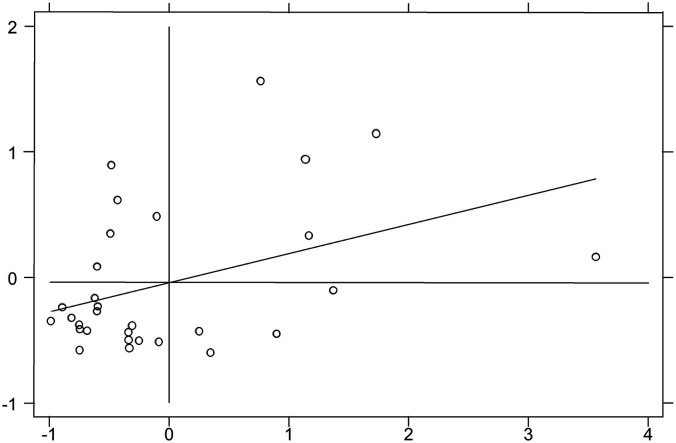
Moran scatter plot in 2010.

**FIGURE 4:**
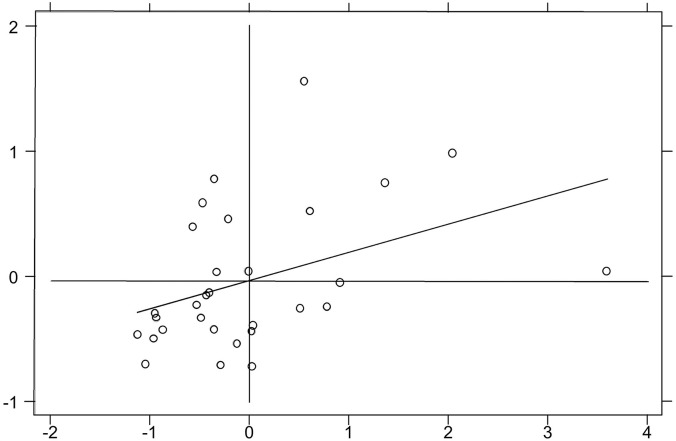
Moran scatter plot in 2014.

**FIGURE 5:**
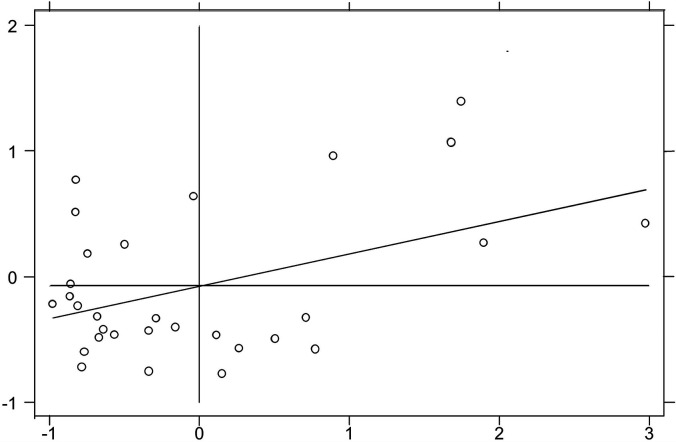
Moran scatter plot in 2016.

Moran scatter plot is often used for local spatial auto-correlation analysis method. Due to the limitation of space, only Moran scatter plots for 2006, 2010, 2014, and 2016 are shown in this paper (Figures 2–5). It can be found that the spatial distribution pattern of SDC is stable in the past 11 years, and most of them are located in the third quadrant (Low-Low). This indicates that most provincial SDC have relatively weak and adjacent, and only a few provinces have strong and adjacent SDC, distributed in the first quadrant (High-High).

### Further Study of Regional Heterogeneity

**FIGURE 6:**
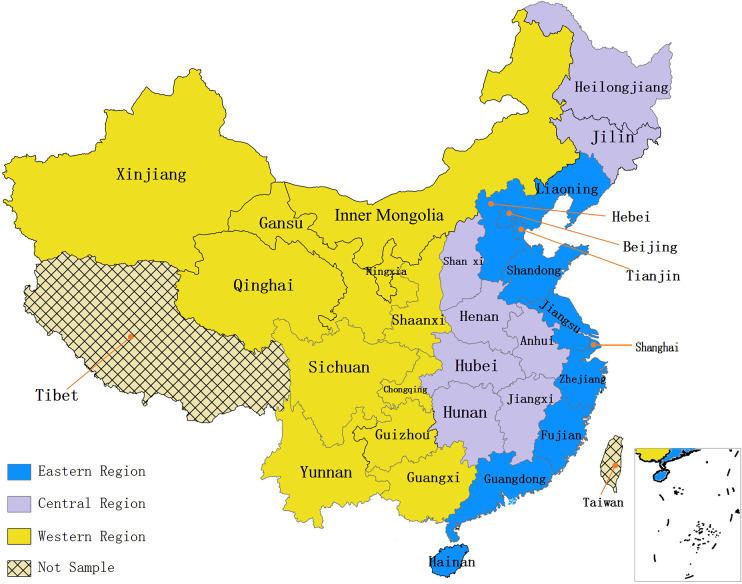
Geographical distribution of eastern, central and western China.

In this paper, regressions of Equation 2 are conducted separately for samples from eastern, central and western China (see Figure 6 for geographical distribution) to further analyze their regional heterogeneity and to test Hypothesis 3. Regressions (I), (II), (III) and (IV) in Table 6 show the results for samples from eastern, central and western China and the whole China, respectively. Among them, the results of (IV) are the same as Table 3 and are presented here as a benchmark for comparison.

**TABLE 6:** Regression results of regional heterogeneity.

**Explanatory variables**	**(I)**	**(II)**	**(III)**	**(IV)**
**Direct effect**				
OCP	0.0185 (0.5248)	0.1038 (0.4545)	1.3552** (0.5870)	0.6608* (0.3776)
FDI	0.4098*** (0.1203)	−0.0090 (0.2583)	0.1027*** (0.0359)	0.1853*** (0.0483)
NR	0.0992** (0.0446)	0.3144*** (0.0258)	0.3705*** (0.0961)	0.3172*** (0.0531)
ER	0.2485 (0.1520)	0.1102 (0.0702)	−0.0553 (0.0774)	0.1075 (0.1198)
**Indirect effect**				
OCP	−0.0194 (0.0930)	−0.0372 (0.1083)	0.0264 (0.3524)	0.0598 (0.1087)
FDI	−0.0231 (0.1478)	0.1576*** (0.0600)	−0.0338 (0.0987)	−0.1031* (0.0602)
NR	−0.0497 (0.0680)	0.0768 (0.1595)	0.5975** (0.2602)	0.3723*** (0.1366)
ER	−0.0349 (0.2067)	0.2793*** (0.0959)	0.4585* (0.2562)	0.4553** (0.2109)
Obs	121	88	121	330
Time-period fixed effect	Yes	Yes	Yes	Yes
Spatial fixed effect	Yes	Yes	Yes	Yes
*R* ^2^	0.5586	0.4293	0.5362	0.5845

***, ** and * indicates statistical significance at the 1%, 5% and 10% levels, respectively.

The regression results from (I) to (IV) reveal that the regression coefficients of OCP, the core explanatory variable of most interest in this paper, change in both significance and coefficient size. The OCP coefficients of the direct effects in eastern and central China do not pass the significance test at a significance level of 10%, indicating that there is no significant relationship between OCP and SDC of the region. However, in western China, the OCP coefficient becomes more significant and the value increases significantly, which indicates that there is a positive relationship between OCP and SDC in western China. The above findings also prove that there is regional heterogeneity in the impact of OCP on SDC (H3 holds).

## Discussion

Table 3 proves that hypothesis H1 holds while hypothesis H2 does not. This indicates that officials’ competitive pressure helps to enhance the sustainable development capacity of their own regions, but does not have a significant effect on their neighboring regions. On the one hand, this finding proves that local officials at the provincial level tend to carry out measures that help to enhance sustainable development capacity (SDC) when they face competitive pressure from promotion, which also shows that the officials’ performance theory view still applies after the environmental indicators are incorporated into the officials’ appraisal system. On the other hand, competition among local officials does not negatively affect neighboring provinces, suggesting that the majority of provincial officials are willing to promote sustainable development, and that their behavior does not cause a “siphon effect” to the detriment of neighboring provinces. Further, Table 6 suggests that the effect of officials’ competitive pressure (OCP) on SDC is regionally heterogeneous, i.e., the effect is only found in western China. In response to this finding, this paper argues that the eastern provinces of China were the first regions to implement sustainable development strategies, and their economic level and ideological advancement are also ahead of other regions. However, there is also less room to improve SDC – at least not by the will of provincial officials and governance preferences. The situation is similar in central China’s provinces. Western Chinese provinces, on the other hand, have great room for development because of their lower levels of SDC. Thus, the governing preferences of local officials under competitive pressure can significantly improve the region’s SDC. In addition, the indirect effects of the OCP coefficients are not significant in either the national sample or the three regional samples. This paper suggests that this is due to the fact that sustainable development has become a “consensus” under the leadership and support of the central government.

The weights in Table 1 indicate only the “degree of variation” and not the “importance.” Analyzing the average weights of the SDC secondary indicators in Table 1, it can be seen that the top four indicators—technology market activity (22.59%), number of patents granted per 10,000 people (14.76%), R&D capital stock (11.50%) and full-time equivalent of R&D personnel (9.80%)—contribute nearly 60% to SDC value. In addition, all four secondary indicators are closely related to technology and innovation, indicating that the differences in SDC among provinces are mainly derived from the innovation dimension. This suggests that the development of the innovation dimension among provinces is extremely uneven, which will hinder the building of sustainable development. Combining this finding, this paper adds to the discussion of the above empirical results. First, the OCP does not negatively affect the SDC of neighboring provinces, and it can be further argued that its behavior does not have a “siphon effect” on innovation resources, indicating that there is no shortage of total innovation resources in China. Second, the effect of OCP on SDC is only effective in western China, which is due to the relative lack of innovation resources in western China and implies that the central government should increase the allocation of innovation resources to the western region.

For other variables: (1) the direct effect of foreign direct investment (FDI) is significant in eastern and western China, but not in central China. This may be due to the fact that FDI brings advanced production technology and management experience, which helps to improve production efficiency and achieve energy saving and emission reduction, thus increasing the SDC of the province. The western region is not affected by this due to the additional allocation from the central government. In addition, there is a positive spillover effect of FDI only in the central region. This may be due to the gradual transfer of secondary industries from developed to less developed neighboring regions, which improves the industrial structure of developed regions and thus enhances the SDC of developed regions. (2) The direct effect of nationalization rate (NR) is significantly positive in all three regions, indicating that the higher the NR, the stronger the SDC of this province. This is consistent with the previous finding that state-owned enterprises (SOEs) will enhance their SDC at any cost. However, there is a positive spillover effect of NR only for the western region, which this paper judges to be due to the higher level of SDC in the eastern and central provinces and the limited radiative impact of SOEs. These regions are in greater need of purely innovative outcomes to raise their SDC levels. (3) The direct effect of environmental regulation (ER) is insignificant in all three regions, suggesting that pure environmental regulation is not sufficient to raise SDC in this province, which also reflects that the Chinese government has been able to better coordinate the relationship between industrial pollution and economic development. Otherwise, high-intensity emission reduction would inevitably affect local economic development and drag down sustainable construction. The indirect effect of ER is significantly positive in both central and western China. This is because environmental regulation has positive externalities, so environmental regulation behavior in this province will benefit neighboring provinces, while this spillover effect is not significant in eastern China due to the generally higher environmental quality.

## Conclusion and Policy Recommendations

This paper mainly explores the role of official competitive pressure (OCP) on sustainable development capacity (SDC) and further analyzes its spillover effects and regional heterogeneity. The empirical results show that: (1) the SDC at the provincial level in China is characterized by positive spatial agglomeration (mainly low-low agglomeration); (2) OCP can only affect the SDC in western provinces of China, while there is no significant spillover effect; (3) foreign direct investment, nationalization rate of enterprises and environmental regulation have their own unique effects on SDC, and such effects have spillover effects. However, there is regional heterogeneity in these effects.

Based on the above findings, this paper proposes the following policy recommendations.

First, the Chinese government’s policy of including environmental indicators in the officials’ assessment system has far-reaching implications for China’s sustainable development, and should be adhered to. At the same time, there is still room for improving this policy. For example, since the effect of OCP to promote SDC is significant in the western provinces, the reward and punishment mechanism can be strengthened to make full use of this effect. For eastern and central China, on the other hand, since officials are already consciously and actively pursuing sustainable development strategies, the corresponding incentive mechanisms could be weakened.

Second, an important dimension that separates provinces’ SDC level is innovation, which should be strengthened by the central government. For example, building cross-provincial innovation exchange platforms to alleviate the local shortage of innovative talents caused by the concentration of talents; promoting industry-university-research cooperation among local governments, research institutions and universities to give full play to the research potential of universities, and to speed up the process of economicization of research results; improving the talent evaluation system and enhancing the treatment of talent training and talent introduction to ensure that innovative talents can be injected into the market.

Third, adjust the FDI introduction strategy and optimize the spatial distribution of FDI. The eastern and western regions can introduce more FDI in high technology and advanced management, while the central region can introduce FDI in a comprehensive manner.

Fourthly, promote the reform of state-owned enterprises (SOEs). Under the premise of ensuring that SOEs’ “fulfill social responsibilities as the main purpose,” efforts should be made to improve the economic competitiveness of SOEs, focusing on solving problems such as redundant management bodies and lack of incentive mechanisms. On the one hand, privatize SOEs in some industries and force them to improve themselves in the market competition and turn losses into profits. On the other hand, maintain the share of SOEs in industries with serious environmental pollution in order to give full play to SOEs’ environmental functions and promote sustainable development.

## Data Availability Statement

The data analyzed in this study is subject to the following licenses/restrictions: The main data comes from CNKI, which can only be obtained by purchasing database. Requests to access these datasets should be directed to HX, 2019110139@email.cufe.edu.cn.

## Author Contributions

HX designed the study, collected data, and performed analysis, interpreted the results under the supervision of KW and GL, and drafted the manuscript. YZ built the framework of the study. KW, GL, and YZ contributed with interpretations and revisions of the manuscript draft. GL reviewed the grammar of the manuscript. All authors were involved in writing the manuscript, critically revising it for important intellectual content, and gave final approval of the version to be published.

## Conflict of Interest

The authors declare that the research was conducted in the absence of any commercial or financial relationships that could be construed as a potential conflict of interest.

## Publisher’s Note

All claims expressed in this article are solely those of the authors and do not necessarily represent those of their affiliated organizations, or those of the publisher, the editors and the reviewers. Any product that may be evaluated in this article, or claim that may be made by its manufacturer, is not guaranteed or endorsed by the publisher.

## Footnotes

1 Officials’ turnover data comes from: http://www.gov.cn/; http://www.hotelaah.com/liren/index.html.
